# Advances in Characterization and Processing of Metallic Materials

**DOI:** 10.3390/ma19142946

**Published:** 2026-07-08

**Authors:** Young-Ok Yoon, Seong-Ho Ha

**Affiliations:** Materials Supply Chain R&D Department, Korea Institute of Industrial Technology, Incheon 21999, Republic of Korea; veryoon@kitech.re.kr

## 1. Introduction and Scope

In recent years, the field of metallic materials has undergone rapid development, driven by increasing demands for high-performance structural and functional materials in industries such as aerospace, automotive, energy, defense, and biomedical engineering [1,2,3]. Recent conflicts have shown a paradigm shift in modern warfare toward unmanned and autonomous systems, while advances in physical artificial intelligence are accelerating the deployment of intelligent robotic platforms. These trends are driving a growing demand for advanced materials suited to meeting increasingly stringent requirements for strength, lightweight design, durability, and functional integration.

A key factor governing the performance of metallic materials is their microstructure, which is strongly influenced by processing history, chemical composition, and service conditions. As a result, understanding and controlling microstructural evolution has become a central focus in modern materials science. Processes such as solidification, additive manufacturing, thermomechanical deformation, and heat treatment play a critical role in determining the final microstructure of metallic systems, which ultimately governs their mechanical, physical, and functional properties through intricate process–microstructure–property relationships [2,4,5]. These processes involve complex physical and chemical phenomena, including phase transformations, diffusion, oxidation, and deformation mechanisms. Consequently, advanced characterization techniques ranging from electron microscopy to computational modeling are essential for elucidating the relationships between process, microstructure, and property.

This Special Issue, entitled “Characterization of Metallic Materials: Solidification, Deformation, Heat Treatment and Other Related Phenomena—Third Edition”, focuses on the characterization of metallic materials under various material conditions, including solidification, forming, and heat treatment. It encompasses a broad range of experimental and analytical techniques, such as transmission electron microscopy, scanning electron microscopy, surface and interface analysis, and numerical simulation methods. Additionally, computational approaches and data-driven techniques are increasingly being integrated into materials research, enabling more accurate prediction and optimization of material behavior.

## 2. Overview of the Published Articles

The papers published in this Special Issue cover a diverse range of metallic materials, including steels, non-ferrous alloys, high-entropy alloys, composites, superalloys, cast irons, and functional alloy systems. Together, they provide valuable insights into the processing, microstructural evolution, characterization, and performance of advanced metallic materials for structural, functional, and engineering applications.

### 2.1. Solidification and Casting Phenomena

Solidification remains one of the most fundamental processes governing the microstructure formation and the resulting properties of metallic materials. Several contributions provide new insights into solidification behavior, microstructural evolution, and defect formation under various processing conditions.

Zhu et al. investigated the solidification behavior of Sm–Co–Fe alloys and clarified the relationship between phase formation and microstructural evolution during casting. Zakaraia et al. indicated that the application of a rotating magnetic field during the solidification of hypereutectic Al–Si alloys significantly modified the distribution and morphology of primary silicon particles, leading to improved structural homogeneity. Del Campo-Castro et al. employed thermal analysis techniques to characterize the solidification behavior of cast irons, highlighting the importance of process monitoring for quality control and defect prevention. The formation of casting defects and process-related heterogeneities was further addressed by Song et al., who examined defect evolution during the continuous casting of stainless steels and proposed approaches for improving process stability. Cao et al. investigated eutectic morphology transitions in Ni–Sn alloys during directional solidification. By combining Bridgman experiments with cellular automaton simulations, they demonstrated that velocity transitions can induce anomalous eutectic formation.

### 2.2. Additive Manufacturing and Rapid Solidification

Additive manufacturing has emerged as a transformative processing route capable of producing complex geometries while simultaneously enabling unprecedented control of microstructure through rapid solidification. Guo et al. studied wire-arc additive manufacturing based on a cold metal transfer (CMT) process for steel/copper bimetallic structures and demonstrated strategies to improve interfacial bonding and microstructural integrity. Chen et al. investigated the microstructural evolution and mechanical behavior of a selective laser melted GH4099 superalloy, revealing the effects of rapid solidification and subsequent thermal history on phase constitution and performance.

### 2.3. Thermomechanical Processing and Deformation Behavior

The optimization of thermomechanical processing routes remains essential for tailoring microstructures and mechanical properties in structural materials. Wang et al. investigated the hot deformation behavior of 6063 aluminum alloy and established constitutive relationships useful for process optimization. Sovetbayev et al. examined the effects of skew rolling on 6082 aluminum alloy and demonstrated its potential for enhancing microstructural refinement and mechanical performance. Zhuo et al. combined equal-channel angular pressing and rolling to modify the microstructure of Zn–Ag–Mg alloys, obtaining significant grain refinement and property enhancement. Baqeri et al. investigated thermomechanical processing strategies for CrVNb microalloyed steel and clarified the role of processing parameters in controlling precipitation and strengthening mechanisms.

### 2.4. Heat Treatment and Microstructural Evolution

Heat treatment processes remain indispensable tools for tailoring material performance and ensuring structural reliability. Li et al. investigated recrystallization behavior in high-entropy alloys and provided insights into grain structure evolution under thermal exposure. Qi et al. examined induction heating processes for bulb flat steel and evaluated the resulting thermal and microstructural responses. Guo et al. studied bainitic steels strengthened by NiAl precipitates, revealing the critical role of precipitation behavior in achieving improved strength-ductility combinations. Zhang et al. analyzed carburized gear steels and clarified the influence of heat treatment on microstructure and mechanical properties. Adamczak-Bugno et al. studied the effects of elevated temperatures on prestressing steel. Their study showed that exposure to temperatures up to 700 °C led to pearlite spheroidization, carbide coarsening, significant strength degradation, and a transition from quasi-brittle to ductile failure mechanisms.

### 2.5. Surface Engineering and Coating Technologies

Surface engineering technologies are increasingly employed to enhance corrosion resistance, wear performance, and service life in demanding environments. Cazac et al. investigated phosphate coating technologies and their effects on surface characteristics and protective performance. Zagula-Yavorska et al. explored aluminide coatings designed for high-temperature applications and evaluated their microstructural stability and oxidation resistance. Park and Lee examined the phosphatability of automotive steels and identified factors influencing coating quality and process effectiveness.

### 2.6. Advanced Characterization, Modeling, and Data-Driven Approaches

The increasing availability of advanced characterization and evaluation techniques, computational tools, and data-driven methodologies is transforming materials research by enabling deeper understanding across multiple length scales. Zhang et al. applied machine learning approaches to amorphous alloy systems and demonstrated the potential of data-driven methodologies for accelerating materials discovery and optimization. Shen et al. utilized high-throughput nanoindentation techniques to efficiently evaluate local mechanical responses and microstructural heterogeneity. Kurdi et al. combined micropillar compression testing with microstructural characterization to investigate deformation mechanisms in high-entropy-alloy-reinforced titanium composites.

Computational modeling also played a important role in several contributions. Cao et al. integrated cellular automaton simulations with directional solidification experiments to reveal the mechanisms governing anomalous eutectic formation in Ni–Sn alloys. Adamczak-Bugno et al. combined acoustic emission monitoring with finite element analysis, establishing correlations between acoustic emission parameters, local strain fields, and stress evolution during the deformation of fire-exposed prestressing steel.

## 3. Conclusions

The contributions shown in this Special Issue collectively demonstrate the importance of microstructural control in determining the properties and performance of metallic materials. Across a wide range of alloy systems and processing techniques, it is evident that even subtle changes in processing parameters can lead to significant variations in microstructural features such as grain size, phase formation and distribution, and defect density. A key trend emerging from these studies is the increasing integration of experimental and computational approaches. Advanced characterization techniques, combined with modeling and simulation, enable a deeper understanding of complex phenomena, including phase transformations, deformation mechanisms, and interface behavior. Furthermore, the development of advanced processing technologies, such as additive manufacturing and severe plastic deformation, provides new opportunities for tailoring microstructures and achieving enhanced material performance. Simultaneously, traditional processes such as casting and heat treatment continue to play a vital role, particularly when optimized using modern analytical tools.
